# Toxins–Useful Biochemical Tools for Leukocyte Research

**DOI:** 10.3390/toxins2040428

**Published:** 2010-03-26

**Authors:** Susana Cubillos, Johannes Norgauer, Katja Lehmann

**Affiliations:** Department of Dermatology, Medical School Jena, Erfurter Strasse 35, 07740, Jena, Germany; Email: susana.cubillos@med.uni-jena.de (S.C.); katja.lehmann@med.uni-jena.de (K.L.)

**Keywords:** toxins, leukocytes, signal transduction

## Abstract

Leukocytes are a heterogeneous group of cells that display differences in anatomic localization, cell surface phenotype, and function. The different subtypes include e.g., granulocytes, monocytes, dendritic cells, T cells, B cells and NK cells. These different cell types represent the cellular component of innate and adaptive immunity. Using certain toxins such as pertussis toxin, cholera toxin or clostridium difficile toxin, the regulatory functions of Gα_i_, Gαs and small GTPases of the Rho family in leukocytes have been reported. A summary of these reports is discussed in this review.

## 1. Introduction

**Figure 1:**
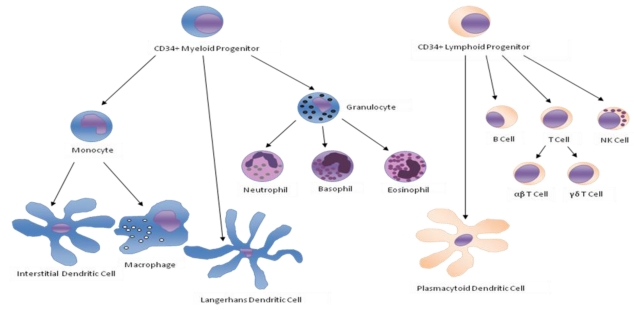
Differentiation diagram of CD34 bone marrow stem cells and progenitors into the different leukocytes subtypes.

Leukocytes are a heterogeneous group of cells that display differences in anatomic localization, cell surface phenotype, and function. They originate from CD34 bone marrow stem cells and progenitors of the different subtypes in the bone marrow. Finally, these progenitors or precursors circulate in the blood or are seeded *via* the bloodstream to the tissues where they give rise to highly specialized cells. The different subtypes include e.g., granulocytes (neutrophils, eosinophils, basophils), monocytes, dendritic cells, T cells, B cells and NK cells (Figure 1). These different cell types represent the cellular component of innate and adaptive immunity, protecting the body against invaders and functioning in tumor surveillance. Neutrophils, eosinophils and monocytes are cellular components of innate immunity and are involved in first line of defense against bacteria and helminth infections. Dendritic cells have the ability to take up antigens and readily degrade them in endocytic vesicles to produce antigenic peptides capable of binding major histocompatibility complex (MHC) class II. In response to danger signals (*i.e.*, tissue damage, pathogen-derived products, or inflammatory cytokines), dendritic cells migrate to lymphoid organs where they interact with CD4+ T cells to initiate specific immune responses. CD4+ T cells are involved in the development and clonal expansion of effector αβ T (e.g., CD8+ cytotoxic T cells) or B cells. NK cells attack cells that are missing "self" markers of MHC class I without prior activation: therefore, they play a major role in the rejection of tumors and cells infected by viruses. In contrast to αβ T cells, γδ T cells do not require antigen processing and MHC presentation of peptide epitopes for recognition of antigens. They are involved in defense against bacteria and in tumor surveillance. In order to fulfill all these different activities, the various subtypes of leukocytes perform highly specialized effector functions, summarized in Table 1.

**Table 1:** The main specific functions of the leukocyte subtypes.

Cell Type	Main functions
Monocytes	Monocytes ingest antibodies or complement opsonized material by **phagocytosis** [1]. They are able to produce reactive oxygen species. Once activated, monocytes can **present antigens** to T cells and secrete **cytokines** such as IL-1, TNF-α, IL-6 and INF-α/β. They can express cell-adhesion molecules and migrate to inflammation sites by **chemotaxis.**
Neutrophils	Neutrophils can express cell-adhesion molecules that allow **diapedesis** [2,3,4] and they can move to a site of infection or inflammation through a process called **chemotaxis** [5]. They can directly attack micro-organisms by **phagocytosis** [6] and are able to produce reactive oxygen species [7], release soluble anti-microbials and granule proteins (**degranulation**), and generate neutrophil extracellular traps (**NETs**) [8].
B Cells	B cells produce **antibodies**, which assist in the destruction of microbes by binding to them and making them easier targets for phagocytes and activation of the complement system, and eventually develop into **memory** B cells after activation by antigen interaction.
αβ T Cells	T cells play a central role in cell-mediated immunity. There are several subgroups:
	T cells that express the CD4 protein on their surface are called **T-helper cells** because they assist B cells and cytotoxic T cells. T-helper cells recognize peptide antigens associated with MHC-class II on the surface of antigen presenting cells (APCs). Once activated, they divide rapidly and secrete **cytokines** that regulate the immune response.
	T cells that express the CD8 protein on their surface are called **T-cytotoxic cells (CTLs)** and can destroy virally infected cells and tumor cells with toxic granules, and are also implicated in transplant rejection.
	**Memory T cells** are a subset of antigen-specific T cells that persist long-term after an infection has resolved. They quickly expand to large numbers of effector T cells upon re-exposure to their cognate antigen, thus providing the immune system with "memory" against past infections.
	**Regulatory T cells**, formerly known as suppressor T cells, are crucial for the maintenance of immunological tolerance. Their major role is to regulate T cell-mediated immunity toward the end of an immune reaction and to suppress auto-reactive T cells that escaped the process of negative selection in the thymus.
γδ T Cells	This small group of T cells possesses a distinct T cell receptor (TCR) on their surface. It seems that these cells are able to recognize whole proteins rather than requiring peptides to be presented by MHC molecules on APCs.
	γδ T cells utilize a variety of different mechanisms to regulate the inflammatory response. They secret **cytokines** and mediate inflammatory balance by inducing **apoptosis** in opposing cell populations (**cytotoxicity**).
NK Cells	NK cells play a major role in the rejection of tumors and cells infected by viruses. The cells kill by **releasing small cytoplasmic granules** of proteins called perforins and granzymes. They can also kill target cells by mean of antibody dependent cell mediated cytotoxicity (**ADCC**).
Dendritic Cells	Upon activation, dendritic cells **phagocyte** and process antigens, **migrate** to lymph nodesto **present the antigens** to antigen-specific T cells and secrete various **cytokines** like IL-12 [9] and INF-γ [10] and **chemokines** that initiate and/or enhance many T and B lymphocyte responses.

Recruitment of leukocytes from the circulation into inflammatory tissues or tumors requires a series of soluble and cell-bound interactions between the responding leukocyte and vascular endothelial barrier. Chemotactic factors are believed to be responsible for this selective adhesion and transmigration. A superfamily of small, soluble, structurally-related molecules called ‘chemokines’ has been identified and shown to selectively promote the rapid adhesion and chemotaxis of a variety of leukocyte subtypes both *in vivo* and *in vitro*. In addition, bacterial wall-derived peptides, lipids, complement fragments as well as micro-milleu components such as ATP, ADP, adenosine, spingosine-1-phosphate, lysophospatidic acid, histamine and serotonin have chemotactic activity for certain subtypes.

Most chemotaxins in leukocytes develop their biological activity by interactions with specific G_i _protein-coupled serpentine receptors (GPCRs). The coupled guanine nucleotide-binding proteins (G proteins) represent a family of heterotrimeric proteins composed of α-, β-, and γ subunits. In humans, there are at least 21 Gα subunits, 6 Gβ subunits and 12 Gγ subunits [11]. The α-subunits are further subdivided in α_i_,α_s, _α_q_ and α_12_ subgroups. Recently, a fifth Gα protein, Gv, was discovered [12]. Heterotrimeric G proteins function as "molecular switches," alternating between an inactive guanosine diphosphate (GDP)- and active guanosine triphosphate (GTP)-bound state in order to regulate downstream signaling pathways and cell processes. They are activated in response to a conformation change in the G protein-coupled receptor after interactions with the specific ligands, followed by the exchange of GDP for GTP on the α-subunits. This results in the dissociation into active GTP-bound α-subunits and free βγ-dimers. These components again activate other proteins in signal transduction pathways. The Gα_i_ subgroup is named in accordance to their inhibitory function on adenylylcyclase. Free βγ-subunits activate phospholipase Cβ2 [13]. This enzyme cleaves phosphatidylinositol (4,5)-bisphosphate (PIP2) to form two second-messenger molecules called inositol triphosphate (IP_3_) and diacylglycerol (DAG). The latter activates another enzyme called protein kinase C (PKC), and IP_3_ triggers the release of calcium from intracellular stores. Free βγ-subunits are also able to activate phosphatidylinositol-3-kinase-γ, which phosporylates PIP_2_ generating PIP_3_. This lipid is able to interact with pleckstrin homology domains (PH-domains) on a broad spectrum of effector proteins such as proteins of the serine/threonine-specific protein kinase family AKT. Once correctly positioned in the membrane *via* binding of PIP_3_, Akt is phosphorylated on twokey residues: T308 in the activation loop by PDK1 [14] and S473 in the hydrophobic motif of the C-terminal. The mammalian target of rapamycin complex 2 (mTORC2) acts as the long-sought PDK2 molecule [15]. Phosphorylation by mTORC2 stimulates the subsequent phosphorylation of Akt by PDK1. Activated Akt can then go on to activate or deactivate its myriad substrates *via* its kinase activity. Besides being a downstream effector of PI 3-kinases, Akt may also be activated in a PI 3-kinase-independent manner. Studies have suggested that cAMP-elevating agents could activate Akt through protein kinase A (PKA), although these studies are disputed and the mechanism of action is unclear. Akt regulates different cellular responses (e.g., cell proliferation, cell survival, and metabolism) by binding and regulating several downstream effectors. Akt can influence cell survival by regulating proapoptotic proteins like the BCL-2 family member BAD [16] or the indirect activation of nuclear factor κB (NF-κB) by regulating IκB kinase (IKK). Akt-mediated phosphorylation of *p53*-specific E3 ubiquitin ligase (MDM2) results in degradation of p53. The inhibitory phophorylating of glycogen synthase kinase 3 (GSK-3) by Akt leads to promotion of glycogen synthesis.

Aktivation of GPCR is also closely associated with the activation Rho family GTPases. Rho/Rac proteins constitute a subgroup of the Ras superfamily of GTP hydrolases. These proteins are classified into six subfamilies: Rho, Rac, Cdc42, Rnd, RhoBTB and RhoT/Miro. The ‘‘molecular switches’’ between the GDP- or GTP-bound state is controlled by two types of regulatory proteins GEFs and GAPs. GEFs induce the exchange of GDP for GTP molecules, promoting the activation of these proteins during signal transduction. The hydrolysis of the bound GTP molecules by GAPs, results in the transfer of the GTPase back to the inactive state. GEFs like P-Rex1 or SWAP70 can be activated by PIP_3_ since they contain the PH domain. The Rho/Rac-GTPases coordinate diverse cellular functions including cytoskeletal events, cell polarity, vesicular trafficking, cell cycle and transcriptomal dynamics. In leukocytes, Rho proteins are essential for epithelial barrier functions, immune cell migration, adhesion, phagocytosis, superoxide production, cytokine secretion and immune cell signaling. This functional diversity can be explained by the interaction of Rac/Rho proteins with many downstream effector molecules. To date, more than 70 potential effectors have been identified e.g., PAK1, Stat3, FlnA (reviewed in [17]).

## 2. Pertussis Toxin

Pertussis toxin (PT) is a protein-based exotoxin produced by the bacterium Bordetella pertussis. It is involved in the colonization of bacteria in the respiratory tract and causes whooping cough. The exotoxin contains six subunits (named S1 through S5 - each complex contains two copies of S4). The subunits are arranged in an A-B structure: the A component is enzymatically active and is formed from the S1 subunit, while the B component is the receptor-binding portion and is made up of subunits S2–S5 [18].

PT is released from Bordetella pertussis in an inactive form. After binding to a cell membrane receptor, it is taken up in an endosome and retrograde transported to the trans-Golgi network and endoplasmic reticulum [19]. At some point during this transport, the A subunit becomes activated, presumably involving the action of glutathione and ATP. PT catalyzes the ADP-ribosylation of Gα_i_ and Gα_o _subunits. This prevents the G proteins from interacting with G protein-coupled receptors on the cell membrane, interrupting intracellular communication. Since the Gα subunits remain in their GDP-bound and inactive state, they cannot inhibit adenylyl cyclase, thereby causing adenylyl cyclase to be inappropriately active and leading to increased cellular concentrations of cAMP within the cell. On the other hand, serpentine receptors interact probably in a very specific manner with a distinct composition of α-, β-, and γ-subunits. Therefore, participation of Gα_i_ in specific receptor-mediated signaling can be also seen in βγ-regulated pathways (e.g., PLCβ or PI3Kγ) due to missing dissociation of the inactive heterotrimeric GDP-bound complex into free βγ-dimers. Using pertussis toxin as a tool, essential participation of Gα_i_ in multiple cell responses and signaling pathways has been reported in leukocytes (Table 2).

**Table 2:** Effect of pertussis toxin on different leukocyte functions. + is an increase or induction, - is an inhibition or decrease of different cell functions.

Cell Type	Cell functions	Pertussis toxin
Monocytes	Phagocytosis	**+/-** [27,28,44]
	Cytokine production	**+/-** [20,23,31]
	Chemotaxis	**-** [21,22,24,30,33]
	Migration	**+** [29,34]
Neutrophils	Cytotoxicity	**-** [25]
	Phagocytosis	**-** [44,45]
	Oxygen reactive species	**-** [39,40,41,42,45]
	Degranulation	**-** [36]
	Migration	**-** [37,43]
	Chemotaxis	**-** [35,38,39,45]
B Cells	Proliferation	**+/-** [49,51,55]
	Activation	**-** [50,53,54,56,57]
	Antibody production	**-** [51]
	Migration	**-** [46,47,48,52]
αβ T Cells	Proliferation	**+** [32,60]
	Cytokine production	**+/-** [60,65,71,72]
	Migration	**-** [61,64,69,70]
	Chemotaxis	**-** [58,62,67,69]
	Th_1_ selection	**+** [66]
	Th_2_ differentiation	**+** [59]
γδ T Cells	Proliferation	**+** [68]
NK Cells	Cytotoxicity	**-** [81]
	Chemotaxis	**-** [73,74,75,76,77,78,79,80,82,83,84]
Dendritic Cells	Antigen presentation	**-** [89]
	Cytokine production	**-** [26,88]
	Migration	**-** [85,86,89]
	Chemotaxis	**-** [63,87,90]

## 3. Cholera Toxin

Cholera toxin is an oligomeric protein complex secreted by *Vibrio cholerae*. It consits of six protein subunits: a single copy of the A subunit (part A), and five copies of the B subunit (part B). The two parts are connected by a disulfide bond. The five B subunits form a five-member ring. The A subunit has two important segments. The A1 portion of the chain is an ADP-ribosyltransferase for Gs proteins, while the alpha helix form of the A2 chain sits in the central pore of the B subunit ring [91].

Cholera toxin bound to GM1 gangliosides on the surface of cell is endocytosed, and transported to the Golgi and the endoplasmatic reticulum as a holotoxin. At the endoplasmatic reticulum, a protein disulfide isomerase unfolds and cut the A1 fragment from the B subunits. Then, at least the A1 subunit is translocated into the cytosol *via* receptor-mediated endocytosis [92]. The free A subunit binds with a partner protein called ADP-ribosylation factor 6, driving a conformation change. Thereafter, the A-subunit catalyses ADP ribosylation from NAD to the Gαs subunit of heterotrimeric G protein, resulting in constitutive cAMP production of the regulatory component of adenylate cyclase. Increased adenylate cyclase activity results in elevated intracellular cyclic AMP (cAMP) production and levels. The pathophysiological consequence of intestinal infection or elevated cAMP levels is therefore secretion of H_2_O, Na^+^, K^+^, Cl^-^, and HCO_3_^-^ into the lumen of the small intestine, resulting in rapid dehydration and diarrhea. Using cholera toxin as a tool, regulatory functions of Gαs proteins and adenylyl cylase in multiple cell responses has been reported in leukocytes (Table 3).

**Table 3:** Effect of cholera toxin on different leukocyte functions. + is an increase or induction, - is an inhibition or decrease, ne means no effect, and the other are simply several Cholera toxin effects on different cell functions.

Cell Type	Cell functions	Cholera toxin
Monocytes	Phagocytosis	**-/**ne [44,112,113]
	Cytokine production	**+/-** [93,94,96,97,98,99,100,101,102,103,109,110,111]
	Oxygen reactive species	ne[108]
	Chemotaxis	ne [105,106,107]
	Migration	ne[104]
Neutrophils	Phagocytosis	**-/**ne [112,119,124,125]
	Oxygen reactive species	**-/**ne [116,117,120,121,123]
	Degranulation	ne [122,123,126]
	Migration	**-/**ne [114,115,126]
	Chemotaxis	**-** [118,127,128,129]
B Cells	Proliferation	**+/-** [131,135,148,149,150,173,179]
	Immunoglobulin production	**+** [130,131,134,135,136,138,142,143,144,147,151,152,155,171]
	Memory cells	**+** [133,141,146,153]
αβ T Cells	Proliferation	**+/-** [131,136,157,162,164,165,167,169,172,175,177,178,179,197]
	Activation	**+/-** [140,143,160,171,172,173,174,176]
	Cytotoxicity	**+** [174]
	Cytokine production	**+/-** [72,78,96,101,154,156,160,161,162,163,164,172]
	Chemotaxis	**-** [183]
	Th_1_ selection	**-** [95,101,159,161,165,166]
	Th_2_ differentiation	**+** [95,101,144,145,156,158,168,170]
	Tolerance or memory	**+** [132,146]
NK Cells	Proliferation	**-** [139]
	Activation	**-** [181,185]
	Cytotoxicity	**-** [81,182,184,186,187]
	Migration	**-** [180]
	Chemotaxis	**-** [83,84,181]
Dendritic Cells	Activation	**+** [194,195,196,200]
	Antigen presentation	**+** [191,197]
	Cytokine production	**+/-** [93,101,161,189,190,192,194,196,199]
	Migration	**+** [137,188,193,199]

## 4. Toxin A and B

Toxin A and Toxin B (TcdA and TcdB) are the major virulenz factors of *Clostridium difficile*. TcdA and Tcd B are exotoxins and belong to the family of clostridial glucosylation toxins. They are very large proteins with a molecular weight between 269 and 308 kDa and are characterized by a modular, tripartide composition [201]. The glucosyltransferase activity of the toxins is located in the N-terminal region. The hydrophobic region in the center of the protein is thought to be involved in the translocation of the toxin from the endosomes into the cytosol of the target cells. The receptor binding seems to be restricted to the C-terminal part. 

Clostridial glucosylating toxins enter eukaryotic target cells according to the ‘short trip model’ of bacterial exotoxin uptake [202]. After binding to the target cell, they are endocytosed. In the endosomal compartment, the toxin undergoes conformational changes allowing the insertion in the endosomal membrane and subsequent pore formation in a pH-dependent manner. After autocatalytical cleavage only the glucosytransferase domain of the N-terminus is released into the cytosol (reviewed by [203]).

**Figure 2:**
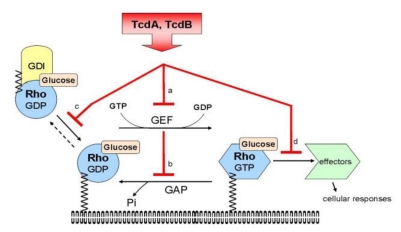
The glucosylation blocks (**a**) the activation of the GTPases by their activators (GEFs) and (**b**) inhibits intrinsic and GAP-stimulated GTPase activity. (**c**) The glucolsylated Rho GTPase is not able to interact with GDI and is therefore located at the plasma membrane. (**d**) The consequence of the glucosylation is the inhibition of the interaction with the effector proteins (kinases or adapter proteins) with subsequent blocking of signal transduction pathways.

The intracellular target of TcdA and TcdB are small GTPases of the Rho family. The toxins modify Rho GTPases *via* mono-O-glycosylation at a threonine residue, which is located in the switch-I region [204]. TcdA and TcdB specifically glucosylate RAC, Cdc42 or Ras [203]. The functional consequence of the glucosylation is diverse, but it always causes the biological inactivity of the GTPases. The glucosylation blocks the activation of the GTPases by their activators (GEFs) and inhibits intrinsic and GAP-stimulated GTPase activity. The glucosylated Rho GTPase is not able to interact with GDI and is therefore located at the plasma membrane. The consequence of the glucosylation is the inhibition of the interaction with the effector proteins (kinases or adaptor proteins) with subsequent blocking of signal transduction pathways (Figure 2). In contrast to endogenous cytosolic mono-O-glucosylation, the glucosylation by TcdA and TcdB seems to be irreversible. 

Inactivation of the Rho proteins leads to drastic changes in eukaryotic cells. The actin cytoskeleton is largely redistributed, accompanied by shrinking, rounding and detachment of the target cells. Using TcdA and TcdB as a tool, several more cellular responses have been reported (Table 4).

**Table 4:** Effect of *Clostridium difficile* Toxin A and Toxin B (TcdA and TcdB) on different leukocyte functions. + is an increase or induction, - is an inhibition or decrease, ne means no effect, and the other are several TcdA and TcdB reported effects on different cell functions.

Cell Type	Cell functions	Clostridium difficile toxins A and B
Monocytes	Phagocytosis	ne [205,214]
	Cytokine production	**+** [206,208,209,210,212,213,214,215]
	Migration	**-** [207,211]
Neutrophils	Phagocytosis	**-/**ne [216,222]
	Oxygen reactive species	**-** [218,219]
	Migration	**+** [209,212,213,219]
	Chemotaxis	**-** [220]
αβ T Cells	Proliferation	**-** [221]
NK Cells	Cytotoxicity	**+** [226]
Dendritic Cells	Maturation	**+** [217]
	Phagocytosis	**-** [224,225]
	Migration	**-** [223]

## 5. Conclusions

Based on the literature using the toxins described here, Gi proteins are involved in processes like cytokine production, chemotaxis, degranulation and production of oxygen reactive species in leukocytes; also in the proliferation and antibody production of lymphocytes; and in the development of Th_1_/Th_2_ immunbalance. On the other hand, Gs proteins limit the cytotoxicity of NK cells, play a major role in Th_1_/Th_2_ balance and development of memory B cells. Finally, the glucosylation and blockade of the small GTPases of the Rho family affects several functions like chemotaxis and oxygen reactive species production of leukocytes, and the proliferation of lymphocytes.

## References

[1] Geske F.J., Monks J., Lehman L., Fadok V.A. (2002). The role of the macrophage in apoptosis: hunter, gatherer, and regulator. Int. J. Hematol..

[2] Lawrence M.B., Springer T.A. (1991). Leukocytes roll on a selectin at physiologic flow rates: distinction from and prerequisite for adhesion through integrins. Cell.

[3] Zimmerman G.A., Prescott S.M., McIntyre T.M. (1992). Endothelial cell interactions with granulocytes: tethering and signaling molecules. Immunol. Today.

[4] Huber A.R., Kunkel S.L., Todd R.F., Weiss S.J. (1991). Regulation of transendothelial neutrophil migration by endogenous interleukin-8. Science.

[5] Zigmond S.H. (1977). Ability of polymorphonuclear leukocytes to orient in gradients of chemotactic factors. J. Cell Biol..

[6] Hirschhorn R., Weissman G. (1965). Isolation and properties of human leukocyte lyosomes *in vitro*. Proc. Soc. Exp. Biol. Med..

[7] Klebanoff S.J. (1968). Myeloperoxidase-halide-hydrogen peroxide antibacterial system. J. Bacteriol..

[8] Volker B., Reichard U., Goosmann C., Fauler B., Uhlemann Y., Weiss D.S., Weinrauch Y., Zychlinsky A. (2004). Neutrophil Extracellular Traps kill Bacteria”. Science.

[9] Reis e Sousa C., Hieny S., Scharton-Kersten T., Jankovic D., Charest H., Germain R.N., Sher A. (1997). *In vivo* microbial stimulation induces rapid CD40 ligand-independent production of interleukin 12 by dendritic cells and their redistribution to T cell areas. J. Exp. Med..

[10] Siega F.P., Kadowaki N., Shodell M., Fitzgerald-Bocarsly P.A., Shah K., Ho S., Antonenko S., Liu Y.J. (1999). The nature of the principal type 1 interferon-producing cells in human blood. Science.

[11] Downes G.B., Gautam N. (1999). The G protein subunit gene families. Genomics.

[12] Oka Y., Saraiva L.R., Kwan Y.Y., Korsching S.I. (2009). The fifth class of Galpha proteins. Proc. Natl. Acad. Sci. USA.

[13] Bonacci T.M., Ghosh M., Malik S., Smrcka A.V. (2005). Regulatory interactions between the amino terminus of G-protein beta gamma subunits and the catalytic domain of phospholipase Cbeta2. J. Biol. Chem..

[14] Scheid M.P., Marignani P.A., Woodgett J.R. (2002). Multiple phosphoinositide 3-kinase-dependent steps in activation of protein kinase B. Mol. Cell Biol..

[15] Sarbassov D.D., Guertin D.A., Ali S.M., Sabatini D.M. (2005). Phosphorylation and regulation of Akt/PKB by the rictor-mTOR complex. Science.

[16] Datta S.R., Dudek H., Tao X., Masters S., Fu H., Gotoh Y., Greenberg M.E. (1997). Akt phosphorylation of BAD couples survival signals to the cell-intrinsic death machinery. Cell.

[17] Bustelo X.R., Sauzeau V., Berenjeno I.M. (2007). GTP-binding proteins of the Rho/Rac family: regulation, effectors and functions *in vivo*. Bioessays.

[18] Stein P.E., Boodhoo A., Armstrong G.D., Cockle S.A., Klein M.H., Read R.J. (1994). The crystal structure of pertussis toxin. Structure.

[19] Plaut R.D., Carbonetti N.H. (2008). Retrograde transport of pertussis toxin in the mammalian cell. Cell Microbiol..

[20] Schepetkin I.A., Kirpotina L.N., Tian J., Khlebnikov A.I., Ye R.D., Quinn M.T. (2008). Identification of novel formyl peptide receptor-like 1 agonists that induce macrophage tumor necrosis factor alpha production. Mol. Pharmacol..

[21] Park K.S., Lee H.Y., Lee S.Y., Kim M.K., Kim S.D., Kim J.M., Yun J., Im D.S., Bae Y.S. (2007). Lysophosphatidylethanolamine stimulates chemotactic migration and cellular invasion in SK-OV3 human ovarian cancer cells: involvement of pertussis toxin-sensitive G-protein coupled receptor. FEBS Lett..

[22] Leach K., Charlton S.J., Strange P.G. (2007). Analysis of second messenger pathways stimulated by different chemokines acting at the chemokine receptor CCR5. Biochem. Pharmacol..

[23] Fan H., Williams D.L., Breuel K.F., Zingarelli B., Teti G., Tempel G.E., Halushka P.V., Cook J.A. (2006). Gi proteins regulate lipopolysaccharide and Staphylococcus aureus induced cytokine production but not (1-3)-beta-D-glucan induced cytokine suppression. Front. Biosci..

[24] Musso T., Cappello P., Stornello S., Ravarino D., Caorsi C., Otero K., Novelli F., Badolato R., Giovarelli M. (2005). IL-10 enhances CCL2 release and chemotaxis induced by CCL16 in human monocytes. Int. J. Immunopathol. Pharmacol..

[25] Bellner L., Thorén F., Nygren E., Liljeqvist J.A., Karlsson A., Eriksson K. (2005). A proinflammatory peptide from herpes simplex virus type 2 glycoprotein G affects neutrophil, monocyte, and NK cell functions. J. Immunol..

[26] Idzko M., Panther E., Sorichter S., Herouy Y., Berod L., Geissler M., Mockenhaupt M., Elsner P., Girolomoni G., Norgauer J. (2004). Characterization of the biological activities of uridine diphosphate in human dendritic cells: Influence on chemotaxis and CXCL8 release. J. Cell. Physiol..

[27] Schaeffer L.M., Weiss A.A. (2001). Pertussis toxin and lipopolysaccharide influence phagocytosis of Bordetella pertussis by human monocytes. Infect. Immun..

[28] Boschwitz J.S., Batanghari J.W., Kedem H., Relman D.A. (1997). Bordetella pertussis infection of human monocytes inhibits antigen-dependent CD4 T cell proliferation. J. Infect. Dis..

[29] Wong W.S., Luk J.M. (1997). Signaling mechanisms of pertussis toxin-induced myelomonocytic cell adhesion: role of tyrosine phosphorylation. Biochem. Biophys. Res. Commun..

[30] Schratzberger P., Wöll E., Reinisch N., Kähler C.M., Wiedermann C.J. (1996). Secretoneurin-induced *in vitro* chemotaxis of human monocytes is inhibited by pertussis toxin and an inhibitor of protein kinase C. Neurosci. Lett..

[31] Torre D., Pugliese A., Tambini R., Speranza F., Zeroli C. (1993). Production and release of tumor necrosis factor alfa, interleukin-1B and interleukin-6 by human mononuclear leukocytes stimulated with pertussis toxin. New Microbiol..

[32] Grenier-Brossette N., Bourget I., Breittmayer J.P., Ferrua B., Fehlmann M., Cousin J.L. (1991). Pertussis toxin-induced mitogenesis in human T lymphocytes. Immunopharmacology.

[33] Verghese M., Uhing R.J., Snyderman R. (1986). A pertussis/choleratoxin-sensitive N protein may mediate chemoattractant receptor signal transduction. Biochem. Biophys. Res. Commun..

[34] Meade B.D., Kind P.D., Manclark C.R. (1984). Lymphocytosis-promoting factor of Bordetella pertussis alters mononuclear phagocyte circulation and response to inflammation. Infect. Immun..

[35] Mariano-Oliveira A., De Freitas M.S., Monteiro R.Q., Barja-Fidalgo C. (2008). Prothrombin fragments containing kringle domains induce migration and activation of human neutrophils. Int J. Biochem. Cell. Biol..

[36] Frasch S.C., Zemski-Berry K., Murphy R.C., Borregaard N., Henson P.M., Bratton D.L. (2007). Lysophospholipids of different classes mobilize neutrophil secretory vesicles and induce redundant signaling through G2A. J. Immunol..

[37] Lokuta M.A., Nuzzi P.A., Huttenlocher A. (2003). Calpain regulates neutrophil chemotaxis. Proc. Natl. Acad. Sci. USA.

[38] Chakraborty A., Hentzen E.R., Seo S.M., Smith C.W. (2003). Granulocyte colony-stimulating factor promotes adhesion of neutrophils. Am. J. Physiol. Cell. Physiol..

[39] Berger M., Budhu S., Lu E., Li Y., Loike D., Silverstein S.C., Loike J.D. (2002). Different G(i)-coupled chemoattractant receptors signal qualitatively different functions in human neutrophils. J. Leukoc. Biol..

[40] Lärfars G., Gyllenhammar H. (1998). Stimulus-dependent transduction mechanisms for nitric oxide release in human polymorphonuclear neutrophil leukocytes. J. Lab. Clin. Med..

[41] Rossi M.A., Di Mauro C., Dianzani M.U. (1993). Action of lipid peroxidation products on phosphoinositide specific phospholipase C. Mol. Aspects Med..

[42] Balazovich K.J., Almeida H.I., Boxer L.A. (1991). Recombinant human G-CSF and GM-CSF prime human neutrophils for superoxide production through different signal transduction mechanisms. J. Lab. Clin. Med..

[43] Bengtsson T. (1990). Correlation between chemotactic peptide-induced changes in chlorotetracycline fluorescence and F-actin content in human neutrophils: a role for membrane-associated calcium in the regulation of actin polymerization?. Exp. Cell Res..

[44] Brown E.J., Newell A.M., Gresham H.D. (1987). Molecular regulation of phagocyte function. Evidence for involvement of a guanosine triphosphate-binding protein in opsonin-mediated phagocytosis by monocytes. J. Immunol..

[45] Lad P.M., Olson C.V., Smiley P.A. (1985). Association of the N-formyl-Met-Leu-Phe receptor in human neutrophils with a GTP-binding protein sensitive to pertussis toxin. Proc. Natl. Acad. Sci. USA.

[46] Rieken S., Herroeder S., Sassmann A., Wallenwein B., Moers A., Offermanns S., Wettschureck N. (2006). Lysophospholipids control integrin-dependent adhesion in splenic B cells through G(i) and G(12)/G(13) family G-proteins but not through G(q)/G(11). J. Biol. Chem..

[47] Itakura A., Szczepanik M., Campos R.A., Paliwal V., Majewska M., Matsuda H., Takatsu K., Askenase P.W. (2005). An hour after immunization peritoneal B-1 cells are activated to migrate to lymphoid organs where within 1 day they produce IgM antibodies that initiate elicitation of contact sensitivity. J. Immunol..

[48] Lo C.G., Lu T.T., Cyster J.G. (2003). Integrin-dependence of lymphocyte entry into the splenic white pulp. J. Exp. Med..

[49] Deehan M.R., Klaus G.G., Holman M.J., Harnett W., Harnett M.M. (1998). MAP-kinase: a second site of G-protein regulation of B-cell activation *via* the antigen receptors. Immunology.

[50] Rosskopf D., Daelman W., Busch S., Schurks M., Hartung K., Kribben A., Michel M.C., Siffert W. (1998). Growth factor-like action of lysophosphatidic acid on human B lymphoblasts. Am. J. Physiol..

[51] Huang R., Cioffi J., Kimberly R., Edberg J., Mayer L. (1995). B cell differentiation factor-induced human B cell maturation: stimulation of intracellular calcium release. Cell. Immunol..

[52] Cyster J.G., Goodnow C.C. (1995). Pertussis toxin inhibits migration of B and T lymphocytes into splenic white pulp cords. J. Exp. Med..

[53] Huang R., Cioffi J., Berg K., London R., Cidon M., Maayani S., Mayer L. (1995). B cell differentiation factor-induced B cell maturation: regulation *via* reduction in cAMP. Cell. Immunol..

[54] Melamed I., Wang G., Roifman C.M. (1992). Antigen receptor-mediated protein tyrosine kinase activity is regulated by a pertussis toxin-sensitive G protein. J. Immunol..

[55] Kolb J.P., Genot E., Petit-Koskas E., Paul-Eugene N., Dugas B. (1990). Effect of bacterial toxins on human B cell activation. I. Mitogenic activity of pertussis toxin. Eur. J. Immunol..

[56] Dziarski R. (1989). Correlation between ribosylation of pertussis toxin substrates and inhibition of peptidoglycan-, muramyl dipeptide- and lipopolysaccharide-induced mitogenic stimulation in B lymphocytes. Eur. J. Immunol..

[57] Jakway J.P., DeFranco A.L. (1986). Pertussis toxin inhibition of B cell and macrophage responses to bacterial lipopolysaccharide. Science.

[58] Schneider O.D., Weiss A.A., Miller W.E. (2009). Pertussis toxin signals through the TCR to initiate cross-desensitization of the chemokine receptor CXCR4. J. Immunol..

[59] Rossi-Paccani S., Benagiano M., Capitani N., Zornetta I., Ladant D., Montecucco C., D'Elios M.M., Baldari C.T. (2009). The adenylate cyclase toxins of Bacillus anthracis and Bordetella pertussis promote Th2 cell development by shaping T cell antigen receptor signaling. PLoS Pathog..

[60] Schneider O.D., Weiss A.A., Miller W.E. (2007). Pertussis toxin utilizes proximal components of the T-cell receptor complex to initiate signal transduction events in T cells. Infect. Immun..

[61] Matsuyuki H., Maeda Y., Yano K., Sugahara K., Chiba K., Kohno T., Igarashi Y. (2006). Involvement of sphingosine 1-phosphate (S1P) receptor type 1 and type 4 in migratory response of mouse T cells toward S1P. Cell. Mol. Immunol..

[62] Smit M.J., Verdijk P., van der Raaij-Helmer E.M., Navis M., Hensbergen P.J., Leurs R., Tensen C.P. (2003). CXCR3-mediated chemotaxis of human T cells is regulated by a Gi- and phospholipase C-dependent pathway and not *via* activation of MEK/p44/p42 MAPK nor Akt/PI-3 kinase. Blood.

[63] Panther E., Idzko M., Corinti S., Ferrari D., Herouy Y., Mockenhaupt M., Dichmann S., Gebicke-Haerter P., Di Virgilio F., Girolomoni G., Norgauer J. (2002). The influence of lysophosphatidic acid on the functions of human dendritic cells. J. Immunol..

[64] Adamson P., Wilbourn B., Etienne-Manneville S., Calder V., Beraud E., Milligan G., Couraud P.O., Greenwood J. (2002). Lymphocyte trafficking through the blood-brain barrier is dependent on endothelial cell heterotrimeric G-protein signaling. FASEB J..

[65] Nanki T., Lipsky P.E. (2001). Stimulation of T-Cell activation by CXCL12/stromal cell derived factor-1 involves a G-protein mediated signaling pathway. Cell. Immunol..

[66] He J., Gurunathan S., Iwasaki A., Ash-Shaheed B., Kelsall B.L. (2000). Primary role for Gi protein signaling in the regulation of interleukin 12 production and the induction of T helper cell type 1 responses. J. Exp. Med..

[67] Sotsios Y., Whittaker G.C., Westwick J., Ward S.G. (1999). The CXC chemokine stromal cell-derived factor activates a Gi-coupled phosphoinositide 3-kinase in T lymphocytes. J. Immunol..

[68] Zorzeto T.Q., Hihashi H.G., da Silva M.T., Carniel Ede F., Dias W.O., Ramalho V.D., Mazzola T.N., Lima S.C., Morcillo A.M., Stephano M.A., Antonio M.A., Zanolli M.d.L., Raw I., Vilela M.M. (2009). Immunogenicity of a whole cell pertussis vaccine with lipopolysaccharide content in infants. Clin. Vaccine Immunol..

[69] del Pozo M.A., Sánchez-Mateos P., Nieto M., Sánchez-Madrid F. (1995). Chemokines regulate cellular polarization and adhesion receptor redistribution during lymphocyte interaction with endothelium and extracellular matrix. Involvement of cAMP signaling pathway. J. Cell. Biol..

[70] Chaffin K.E., Perlmutter R.M. (1991). A pertussis toxin-sensitive process controls thymocyte emigration. Eur. J. Immunol..

[71] Stanley J.B., Gorczynski R.M., Delovitch T.L., Mills G.B. (1989). IL-2 secretion is pertussis toxin sensitive in a T lymphocyte hybridoma. J. Immunol..

[72] Gilmore W., Weiner L.P. (1988). The effects of pertussis toxin and cholera toxin on mitogen-induced interleukin-2 production: evidence for G protein involvement in signal transduction. Cell. Immunol..

[73] Kim S.D., Kim J.M., Jo S.H., Lee H.Y., Lee S.Y., Shim J.W., Seo S.K., Yun J., Bae Y.S. (2009). Functional expression of formyl peptide receptor family in human NK cells. J. Immunol..

[74] Starnes T., Rasila K.K., Robertson M.J., Brahmi Z., Dahl R., Christopherson K., Hromas R. (2006). The chemokine CXCL14 (BRAK) stimulates activated NK cell migration: implications for the downregulation of CXCL14 in malignancy. Exp. Hematol..

[75] Dagan-Berger M., Feniger-Barish R., Avniel S., Wald H., Galun E., Grabovsky V., Alon R., Nagler A., Ben-Baruch A., Peled A. (2006). Role of CXCR3 carboxyl terminus and third intracellular loop in receptor-mediated migration, adhesion and internalization in response to CXCL11. Blood.

[76] Jin Y., Knudsen E., Wang L., Maghazachi A.A. (2003). Lysophosphatidic acid induces human natural killer cell chemotaxis and intracellular calcium mobilization. Eur. J. Immunol..

[77] Kveberg L., Bryceson Y., Inngjerdingen M., Rolstad B., Maghazachi A.A. (2002). Sphingosine 1 phosphate induces the chemotaxis of human natural killer cells. Role for heterotrimeric G proteins and phosphoinositide 3 kinases. Eur. J. Immunol..

[78] Inngjerdingen M., Damaj B., Maghazachi A.A. (2000). Human NK cells express CC chemokine receptors 4 and 8 and respond to thymus and activation-regulated chemokine, macrophage-derived chemokine, and I-309. J. Immunol..

[79] Imai T., Hieshima K., Haskell C., Baba M., Nagira M., Nishimura M., Kakizaki M., Takagi S., Nomiyama H., Schall T.J., Yoshie O. (1997). Identification and molecular characterization of fractalkine receptor CX3CR1, which mediates both leukocyte migration and adhesion. Cell.

[80] Maghazachi A.A., Skalhegg B.S., Rolstad B., Al-Aoukaty A. (1997). Interferon-inducible protein-10 and lymphotactin induce the chemotaxis and mobilization of intracellular calcium in natural killer cells through pertussis toxin-sensitive and -insensitive heterotrimeric G-proteins. FASEB J..

[81] Maghazachi A.A., Al-Aoukaty A., Naper C., Torgersen K.M., Rolstad B. (1996). Preferential involvement of Go and Gz proteins in mediating rat natural killer cell lysis of allogeneic and tumor target cells. J. Immunol..

[82] Allavena P., Bianchi G., Zhou D., van Damme J., Jílek P., Sozzani S., Mantovani A. (1994). Induction of natural killer cell migration by monocyte chemotactic protein-1; -2 and -3. Eur. J. Immunol..

[83] Maghazachi A.A., al-Aoukaty A., Schall T.J. (1994). C-C chemokines induce the chemotaxis of NK and IL-2-activated NK cells. Role for G proteins. J. Immunol..

[84] Sebok K., Woodside D., al-Aoukaty A., Ho A.D., Gluck S., Maghazachi A.A. (1993). IL-8 induces the locomotion of human IL-2-activated natural killer cells. Involvement of a guanine nucleotide binding (Go) protein. J. Immunol..

[85] Salogni L., Musso T., Bosisio D., Mirolo M., Jala V.R., Haribabu B., Locati M., Sozzani S. (2009). Activin A induces dendritic cell migration through the polarized release of CXC chemokine ligands 12 and 14. Blood.

[86] Lambert H., Hitziger N., Dellacasa I., Svensson M., Barragan A. (2006). Induction of dendritic cell migration upon Toxoplasma gondii infection potentiates parasite dissemination. Cell. Microbiol..

[87] Shin E.H., Lee H.Y., Bae Y.S. (2006). Leukotriene B4 stimulates human monocyte-derived dendritic cell chemotaxis. Biochem. Biophys. Res. Commun..

[88] Oz-Arslan D., Rüscher W., Myrtek D., Ziemer M., Jin Y., Damaj B.B., Sorichter S., Idzko M., Norgauer J., Maghazachi A.A. (2006). IL-6 and IL-8 release is mediated *via* multiple signaling pathways after stimulating dendritic cells with lysophospholipids. J. Leukoc. Biol..

[89] Idzko M., Dichmann S., Ferrari D., Di Virgilio F., la Sala A., Girolomoni G., Panther E., Norgauer J. (2002). Nucleotides induce chemotaxis and actin polymerization in immature but not mature human dendritic cells *via* activation of pertussis toxin-sensitive P2y receptors. Blood.

[90] Idzko M., Panther E., Corinti S., Morelli A., Ferrari D., Herouy Y., Dichmann S., Mockenhaupt M., Gebicke-Haerter P., Di Virgilio F., Girolomoni G., Norgauer J. (2002). Sphingosine 1-phosphate induces chemotaxis of immature and modulates cytokine-release in mature human dendritic cells for emergence of Th2 immune responses. FASEB J..

[91] Merritt E.A., Sarfaty S., van den Akker F., L'Hoir C., Martial J.A., Hol W.G. (1994). Crystal structure of cholera toxin B-pentamer bound to receptor GM1 pentasaccharide. Protein Sci..

[92] Fujinaga Y. (2006). Transport of bacterial toxins into target cells: pathways followed by cholera toxin and botulinum progenitor toxin. J. Biochem..

[93] la Sala A., He J., Laricchia-Robbio L., Gorini S., Iwasaki A., Braun M., Yap G., Sher A., Ozato K., Kelsall B. (2009). Cholera toxin inhibits IL-12 production and CD8alpha+ dendritic cell differentiation by cAMP-mediated inhibition of IRF8 function. J. Exp. Med..

[94] Domingos M.O., Andrade R.G., Barbaro K.C., Borges M.M., Lewis D.J., New R.R. (2009). Influence of the A and B subunits of cholera toxin (CT) and Escherichia coli toxin (LT) on TNF-alpha release from macrophages. Toxicon.

[95] Royaee A.R., Mendis C., Das R., Jett M., Yang D.C. (2006). Cholera toxin induced gene expression alterations. Mol. Immunol..

[96] Royaee A.R., Hammamieh R., Mendis C., Das R., Jett M., Yang D.C.H. (2006). Induction of immunomodulator transcriptional responses by cholera toxin. Mol. Immunol..

[97] Burkart V., Kim Y.E., Hartmann B., Ghiea I., Syldath U., Kauer M., Fingberg W., Hanifi-Moghaddam P., Müller S., Kolb H. (2002). Cholera toxin B pretreatment of macrophages and monocytes diminishes their proinflammatory responsiveness to lipopolysaccharide. J. Immunol..

[98] Seldon P.M., Giembycz M.A. (2001). Suppression of granulocyte/macrophage colony-stimulating factor release from human monocytes by cyclic AMP-elevating drugs: role of interleukin-10. Br. J. Pharmacol..

[99] Burkart V., Kim Y., Kauer M., Kolb H. (1999). Induction of tolerance in macrophages by cholera toxin B chain. Pathobiology.

[100] Yan Z., Yang D.C., Jett M. (1999). Cholera toxin induces tumor necrosis factor alpha production in human monocytes. Mol. Cell. Biol. Res. Commun..

[101] Braun M.C., He J., Wu C.Y., Kelsall B.L. (1999). Cholera toxin suppresses interleukin (IL)-12 production and IL-12 receptor beta1 and beta2 chain expression. J. Exp. Med..

[102] López-Zabalza M.J., Martínez-Lausín S., Bengoechea-Alonso M.T., López-Moratalla N., González A., Santiago E. (1997). Signaling pathway triggered by a short immunomodulating peptide on human monocytes. Arch. Biochem. Biophys..

[103] Mattsson E., Van Dijk H., Van Kessel K., Verhoef J., Fleer A., Rollof J. (1996). Intracellular pathways involved in tumor necrosis factor-alpha release by human monocytes on stimulation with lipopolysaccharide or staphylococcal peptidoglycan are partly similar. J. Infect. Dis..

[104] Zhou D., Luini W., Bernasconi S., Diomede L., Salmona M., Mantovani A., Sozzani S. (1995). Phosphatidic acid and lysophosphatidic acid induce haptotactic migration of human monocytes. J. Biol. Chem..

[105] Badolato R., Johnston J.A., Wang J.M., McVicar D., Xu L.L., Oppenheim J.J., Kelvin D.J. (1995). Serum amyloid A induces calcium mobilization and chemotaxis of human monocytes by activating a pertussis toxin-sensitive signaling pathway. J. Immunol..

[106] Mansfield P.J., Suchard S.J. (1994). Thrombospondin promotes chemotaxis and haptotaxis of human peripheral blood monocytes. J. Immunol..

[107] Sozzani S., Zhou D., Locati M., Rieppi M., Proost P., Magazin M., Vita N., van Damme J., Mantovani A. (1994). Receptors and transduction pathways for monocyte chemotactic protein-2 and monocyte chemotactic protein-3. Similarities and differences with MCP-1. J. Immunol..

[108] Puri J., Taplits M., Alava M., Bonvini E., Hoffman T. (1992). Inhibition of release of arachidonic acid, superoxide and IL-1 from human monocytes by monoclonal anti-HLA class II antibodies: effects at proximal and distal points of inositol phospholipid hydrolysis pathway. Inflammation.

[109] Sung S.S., Walters J.A. (1991). Increased cyclic AMP levels enhance IL-1 alpha and IL-1 beta mRNA expression and protein production in human myelomonocytic cell lines and monocytes. J. Clin. Invest..

[110] Vellenga E., van der Vinne B., De Wolf J.T., Halie M.R. (1991). Simultaneous expression and regulation of G-CSF and IL-6 mRNA in adherent human monocytes and fibroblasts. Br. J. Haematol..

[111] Kassis S., Lee J.C., Hanna N. (1989). Effects of prostaglandins and cAMP levels on monocyte IL-1 production. Agents Actions.

[112] Yang K.D., Augustine N.H., Shaio M.F., Bohnsack J.F., Hill H.R. (1994). Effects of fibronectin on actin organization and respiratory burst activity in neutrophils, monocytes, and macrophages. J. Cell. Physiol..

[113] O'Dorisio M.S., Vandenbark G.R., LoBuglio A.F. (1979). Human monocyte killing of Staphylococcus aureus: modulation by agonists of cyclic adenosine 3',5'-monophosphate and cyclic guanosine 3',5'-monophosphate. Infect. Immun..

[114] Viana C.F., Melo D.H., Carneiro-Filho B.A., Michelin M.A., Brito G.A., Cunha F.Q., Lima AA., Ribeiro R.A. (2002). Pro-inflammatory effects of cholera toxin: role of tumor necrosis factor alpha. Toxicon.

[115] Garcia J.G., Verin A.D., Herenyiova M., English D. (1998). Adherent neutrophils activate endothelial myosin light chain kinase: role in transendothelial migration. J. Appl. Physiol..

[116] Tithof P.K., Watts S., Ganey P.E. (1997). Protein tyrosine kinase involvement in the production of superoxide anion by neutrophils exposed to Aroclor 1242, a mixture of polychlorinated biphenyls. Biochem. Pharmacol..

[117] Carreras M.C., Riobó N.A., Pargament G.A., Boveris A., Poderoso J.J. (1997). Effects of respiratory burst inhibitors on nitric oxide production by human neutrophils. Free Radic. Res..

[118] Suchard S.J., Mansfield P.J. (1996). Neutrophil thrombospondin receptors are linked to GTP-binding proteins. J. Cell. Physiol..

[119] Jin G.F., Guo Y.S., Ball C., Houston C.W. (1993). Insulin-like growth factors enhance phagocytosis by human neutrophils *in vitro*. Regul. Pept..

[120] Braquet P., Hosford D., Koltz P., Guilbaud J., Paubert-Braquet M. (1991). Effect of platelet-activating factor on tumor necrosis factor-induced superoxide generation from human neutrophils. Possible involvement of G proteins. Lipids.

[121] Paubert-Braquet M., Hosford D., Klotz P., Guilbaud J., Braquet P. (1990). Tumor necrosis factor alpha 'primes' the platelet-activating factor-induced superoxide production by human neutrophils: possible involvement of G proteins. J. Lipid Mediat..

[122] Gabler W.L., Creamer H.R., Bullock W.W. (1989). Fluoride activation of neutrophils: similarities to formylmethionyl-leucyl-phenylalanine. Inflammation.

[123] Blackburn W.D., Heck L.W. (1988). Neutrophil activation by surface bound IgG: pertussis toxin insensitive activation. Biochem. Biophys. Res. Commun..

[124] Gresham H.D., Clement L.T., Volanakis J.E., Brown E.J. (1987). Cholera toxin and pertussis toxin regulate the Fc receptor-mediated phagocytic response of human neutrophils in a manner analogous to regulation by monoclonal antibody 1C2. J. Immunol..

[125] Lad P.M., Olson C.V., Grewal I.S. (1986). A step sensitive to pertussis toxin and phorbol ester in human neutrophils regulates chemotaxis and capping but not phagocytosis. FEBS Lett..

[126] Shefcyk J., Yassin R., Volpi M., Molski T.F., Naccache P.H., Munoz J.J., Becker E.L., Feinstein M.B., Sha'afi R.I. (1985). Pertussis but not cholera toxin inhibits the stimulated increase in actin association with the cytoskeleton in rabbit neutrophils: role of the "G proteins" in stimulus-response coupling. Biochem. Biophys. Res. Commun..

[127] Roch-Arveiller M., Boquet P., Bradshaw D., Giroud J.P. (1979). Inhibition by cholera toxin of rat polymorphonuclear leukocyte chemotaxis demonstrated *in vitro* and *in vivo*. Infect. Immun..

[128] Issekutz A.C., Ng M., Biggar W.D. (1979). Effect of cyclic adenosine 3',5'-monophosphate antagonists on endotoxin-induced inhibition of human neutrophil chemotaxis. Infect. Immun..

[129] Rivkin I., Rosenblatt J., Becker E.L. (1975). The role of cyclic AMP in the chemotactic responsiveness and spontaneous motility of rabbit peritoneal neutrophils. The inhibition of neutrophil movement and the elevation of cyclic AMP levels by catecholamines, prostaglandins, theophylline and cholera toxin. J. Immunol..

[130] Smits H.H., Gloudemans A.K., van Nimwegen M., Willart M.A., Soullié T., Muskens F., de Jong E.C., Boon L., Pilette C., Johansen F.E., Hoogsteden H.C., Hammad H., Lambrecht B.N. (2009). Cholera toxin B suppresses allergic inflammation through induction of secretory IgA. Mucosal. Immunol..

[131] Negri D.R., Pinto D., Vendetti S., Patrizio M., Sanchez M., Riccomi A., Ruggiero P., Del Giudice G., De Magistris M.T. (2009). Cholera toxin and Escherichia coli heat-labile enterotoxin, but not their nontoxic counterparts, improve the antigen-presenting cell function of human B lymphocytes. Infect. Immun..

[132] Sun J.B., Flach C.F., Czerkinsky C., Holmgren J. (2008). B lymphocytes promote expansion of regulatory T cells in oral tolerance: powerful induction by antigen coupled to cholera toxin B subunit. J. Immunol..

[133] Jayasekera C.R., Harris J.B., Bhuiyan S., Chowdhury F., Khan A.I., Faruque A.S., Larocque R.C., Ryan E.T., Ahmed R., Qadri F., Calderwood S.B. (2008). Cholera toxin-specific memory B cell responses are induced in patients with dehydrating diarrhea caused by Vibrio cholerae O1. J. Infect. Dis..

[134] Kataoka K., Fujihashi K., Sekine S., Fukuiwa T., Kobayashi R., Suzuki H., Nagata H., Takatsu K., Shizukuishi S., McGhee J.R., Fujihashi K. (2007). Nasal cholera toxin elicits IL-5 and IL-5 receptor alpha-chain expressing B-1a B cells for innate mucosal IgA antibody responses. J. Immunol..

[135] Arce S., Nawar H.F., Muehlinghaus G., Russell M.W., Connell T.D. (2007). *In vitro* induction of immunoglobulin A (IgA)- and IgM-secreting plasma blasts by cholera toxin depends on T-cell help and is mediated by CD154 up-regulation and inhibition of gamma interferon synthesis. Infect. Immun..

[136] Arce S., Nawar H.F., Russell M.W., Connell T.D. (2005). Differential binding of Escherichia coli enterotoxins LT-IIa and LT-IIb and of cholera toxin elicits differences in apoptosis, proliferation, and activation of lymphoid cells. Infect. Immun..

[137] Shreedhar V.K., Kelsall B.L., Neutra M.R. (2003). Cholera toxin induces migration of dendritic cells from the subepithelial dome region to T- and B-cell areas of Peyer's patches. Infect. Immun..

[138] Fan J.L., Peterson J.W., Prabhakar B.S. (2000). Adjuvant effects of cholera toxin b subunit on immune response to recombinant thyrotropin receptor in mice. J. Autoimmun..

[139] Miller J.S., Cervenka T., Lund J., Okazaki I.J., Moss J. (1999). Purine metabolites suppress proliferation of human NK cells through a lineage-specific purine receptor. J. Immunol..

[140] Iijima H., Takahashi I., Hiroi T., Shimaoka M., Kawano S., Nagano K., Hori M., Kiyono H. (1998). Orally administered cholera toxin prevents murine intestinal T cells from staphylococcal enterotoxin B-induced anergy. Gastroenterology.

[141] Benedetti R., Lev P., Massouh E., Fló J. (1998). Long-term antibodies after an oral immunization with cholera toxin are synthesized in the bone marrow and may play a role in the regulation of memory B-cell maintenance at systemic and mucosal sites. Res. Immunol..

[142] Kim P.H., Eckmann L., Lee W.J., Han W., Kagnoff M.F. (1998). Cholera toxin and cholera toxin B subunit induce IgA switching through the action of TGF-beta 1. J. Immunol..

[143] Sverremark E., Fernandez C. (1997). Immunogenicity of bacterial carbohydrates: cholera toxin modulates the immune response against dextran B512. Immunology.

[144] Xu-Amano J., Jackson R.J., Fujihashi K., Kiyono H., Staats H.F., McGhee J.R. (1994). Helper Th1 and Th2 cell responses following mucosal or systemic immunization with cholera toxin. Vaccine.

[145] Xu-Amano J., Kiyono H., Jackson R.J., Staats H.F., Fujihashi K., Burrows P.D., Elson C.O., Pillai S., McGhee J.R. (1993). Helper T cell subsets for immunoglobulin A responses: oral immunization with tetanus toxoid and cholera toxin as adjuvant selectively induces Th2 cells in mucosa associated tissues. J. Exp. Med..

[146] Vajdy M., Lycke N. (1993). Stimulation of antigen-specific T- and B-cell memory in local as well as systemic lymphoid tissues following oral immunization with cholera toxin adjuvant. Immunology.

[147] Nilssen D.E., Friman V., Theman K., Björkander J., Kilander A., Holmgren J., Hanson L.A., Brandtzaeg P. (1993). B-cell activation in duodenal mucosa after oral cholera vaccination in IgA deficient subjects with or without IgG subclass deficiency. Scand. J. Immunol..

[148] Garrone P., Banchereau J. (1993). Agonistic and antagonistic effects of cholera toxin on human B lymphocyte proliferation. Mol. Immunol..

[149] Anastassiou E.D., Yamada H., Boumpas D.T., Tsokos G.C., Thyphronitis G., Balow J., Mond J.J. (1992). Cholera toxin promotes the proliferation of anti-mu antibody-prestimulated human B cells. Cell. Immunol..

[150] Dugas B., Paul-Eugène N., Génot E., Mencia-Huerta J.M., Braquet P., Kolb J.P. (1991). Effect of bacterial toxins on human B cell activation. II. Mitogenic activity of the B subunit of cholera toxin. Eur. J. Immunol..

[151] Lycke N., Severinson E., Strober W. (1990). Cholera toxin acts synergistically with IL-4 to promote IgG1 switch differentiation. J. Immunol..

[152] Chen K.S., Strober W. (1990). Cholera holotoxin and its B subunit enhance Peyer's patch B cell responses induced by orally administered influenza virus: disproportionate cholera toxin enhancement of the IgA B cell response. Eur. J. Immunol..

[153] Lycke N., Hellström U., Holmgren J. (1987). Circulating cholera antitoxin memory cells in the blood one year after oral cholera vaccination in humans. Scand. J. Immunol..

[154] Lee J.B., Jang J.E., Song M.K., Chang J. (2009). Intranasal delivery of cholera toxin induces th17-dominated T-cell response to bystander antigens. PLoS One.

[155] Nyström-Asklin J., Adamsson J., Harandi A.M. (2008). The adjuvant effect of CpG oligodeoxynucleotide linked to the non-toxic B subunit of cholera toxin for induction of immunity against H. pylori in mice. Scand. J. Immunol..

[156] Huang C.F., Wang C.C., Wu T.C., Chu C.H., Peng H.J. (2007). Effect of sublingual administration with a native or denatured protein allergen and adjuvant CpG oligodeoxynucleotides or cholera toxin on systemic T(H)2 immune responses and mucosal immunity in mice. Ann. Allergy Asthma. Immunol..

[157] Vendetti S., Riccomi A., Sacchi A., Sciaraffia E., Gatta L., Pioli C., De Magistris M.T. (2008). Inhibition of T cell proliferation by cholera toxin involves the modulation of costimulatory molecules CTLA-4 and CD28. Immunol. Lett..

[158] Lysaght J., Jarnicki A.G., Mills K.H. (2007). Reciprocal effects of Th1 and Treg cell inducing pathogen-associated immunomodulatory molecules on anti-tumor immunity. Cancer Immunol. Immunother..

[159] Su S.B., Silver P.B., Wang P., Chan C.C., Caspi R.R. (2004). Cholera toxin prevents Th1-mediated autoimmune disease by inducing immune deviation. J. Immunol..

[160] Wang M., Bregenholt S., Petersen J.S. (2003). The cholera toxin B subunit directly costimulates antigen-primed CD4+ T cells *ex vivo*. Scand. J. Immunol..

[161] Lavelle E.C., McNeela E., Armstrong M.E., Leavy O., Higgins S.C., Mills K.H. (2003). Cholera toxin promotes the induction of regulatory T cells specific for bystander antigens by modulating dendritic cell activation. J. Immunol..

[162] Staples K.J., Bergmann M., Tomita K., Houslay M.D., McPhee I., Barnes P.J., Giembycz M.A., Newton R. (2001). Adenosine 3',5'-cyclic monophosphate (cAMP)-dependent inhibition of IL-5 from human T lymphocytes is not mediated by the cAMP-dependent protein kinase A. J. Immunol..

[163] Pacheco S.E., Gibbs R.A., Ansari-Lari A., Rogers P. (2000). Intranasal immunization with HIV reverse transcriptase: effect of dose in the induction of helper T cell type 1 and 2 immunity. AIDS Res. Hum. Retroviruses.

[164] Eriksson K., Nordström I., Czerkinsky C., Holmgren J. (2000). Differential effect of cholera toxin on CD45RA+ and CD45RO+ T cells: specific inhibition of cytokine production but not proliferation of human naive T cells. Clin. Exp. Immunol..

[165] Yamamoto M., Kiyono H., Yamamoto S., Batanero E., Kweon M.N., Otake S., Azuma M., Takeda Y., McGhee J.R. (1999). Direct effects on antigen-presenting cells and T lymphocytes explain the adjuvanticity of a nontoxic cholera toxin mutant. J. Immunol..

[166] McSorley S.J., Rask C., Pichot R., Julia V., Czerkinsky C., Glaichenhaus N. (1998). Selective tolerization of Th1-like cells after nasal administration of a cholera toxoid-LACK conjugate. Eur. J. Immunol..

[167] Sobel D.O., Yankelevich B., Goyal D., Nelson D., Mazumder A. (1998). The B-subunit of cholera toxin induces immunoregulatory cells and prevents diabetes in the NOD mouse. Diabetes.

[168] Marinaro M., Boyaka P.N., Finkelman F.D., Kiyono H., Jackson R.J., Jirillo E., McGhee J.R. (1997). Oral but not parenteral interleukin (IL)-12 redirects T helper 2 (Th2)-type responses to an oral vaccine without altering mucosal IgA responses. J. Exp. Med..

[169] Yankelevich B., Soldatenkov V.A., Hodgson J., Polotsky A.J., Creswell K., Mazumder A.  (1996). Differential induction of programmed cell death in CD8+ and CD4+ T cells by the B subunit of cholera toxin. Cell. Immunol..

[170] Marinaro M., Staats H.F., Hiroi T., Jackson R.J., Coste M., Boyaka P.N., Okahashi N., Yamamoto M., Kiyono H., Bluethmann H., Fujihashi K., McGhee J.R. (1995). Mucosal adjuvant effect of cholera toxin in mice results from induction of T helper 2 (Th2) cells and IL-4. J. Immunol..

[171] Bowen J.C., Nair S.K., Reddy R., Rouse B.T. (1994). Cholera toxin acts as a potent adjuvant for the induction of cytotoxic T-lymphocyte responses with non-replicating antigens. Immunology.

[172] Hörnquist E., Lycke N. (1993). Cholera toxin adjuvant greatly promotes antigen priming of T cells. Eur. J. Immunol..

[173] Woogen S.D., Turo K., Dieleman L.A., Beagley K.W., Elson C.O. (1993). Inhibition of murine T cell activation by cholera toxin B subunit is not mediated through the phosphatidylinositol second messenger system. J. Immunol..

[174] Valitutti S., Dessing M., Lanzavecchia A. (1993). Role of cAMP in regulating cytotoxic T lymphocyte adhesion and motility. Eur. J. Immunol..

[175] Maghazachi A.A. (1992). Cholera toxin inhibits interleukin-2-induced, but enhances pertussis toxin-induced T-cell proliferation: regulation by cyclic nucleotides. Immunology.

[176] Gupta S., Shimizu M., Ohira K., Vayuvegula B. (1991). T cell activation *via* the T cell receptor: a comparison between WT31 (defining alpha/beta TcR)-induced and anti-CD3-induced activation of human T lymphocytes. Cell. Immunol..

[177] Anderson D.L., Tsoukas C.D. (1989). Cholera toxin inhibits resting human T cell activation *via* a cAMP-independent pathway. J. Immunol..

[178] Bjorndahl J.M., Sung S.S., Hansen J.A., Fu S.M. (1989). Human T cell activation: differential response to anti-CD28 as compared to anti-CD3 monoclonal antibodies. Eur. J. Immunol..

[179] Lycke N., Bromander A.K., Ekman L., Karlsson U., Holmgren J. (1989). Cellular basis of immunomodulation by cholera toxin *in vitro* with possible association to the adjuvant function *in vivo*. J. Immunol..

[180] Poggi A., Spada F., Costa P., Tomasello E., Revello V., Pella N., Zocchi M.R., Moretta L. (1996). Dissection of lymphocyte function-associated antigen 1-dependent adhesion and signal transduction in human natural killer cells shown by the use of cholera or pertussis toxin. Eur. J. Immunol..

[181] Maghazachi A.A., al-Aoukaty A. (1993). Guanine nucleotide binding proteins mediate the chemotactic signal of transforming growth factor-beta 1 in rat IL-2 activated natural killer cells. Int. Immunol..

[182] Watanabe M., Kiyohara T., Murata K., Yodoi J., Itakura H. (1993). Cholera toxin inhibits lethal hit stage of natural killer cell-mediated cytotoxicity. Microbiol. Immunol..

[183] Maghazachi A.A. (1991). Tumor necrosis factor-alpha is chemokinetic for lymphokine-activated killer cells: regulation by cyclic adenosine monophosphate. J. Leukoc. Biol..

[184] Whalen M.M., Bankhurst A.D. (1990). Effects of beta-adrenergic receptor activation, cholera toxin and forskolin on human natural killer cell function. Biochem. J..

[185] Hussain A., Himeno K., Mayumi H., Kawamura I., Tsuru S., Nomoto K. (1989). Immunomodulatory effects of cholera toxin in mice. Nat. Immun. Cell. Growth Regul..

[186] Fuyama S., Naiki M., Sendo F. (1982). The mechanism of cholera toxin-induced suppression of natural killer cytotoxicity. Gann..

[187] Fuyama S., Sendo F., Watabe S., Seiji K., Arai S. (1981). Inhibition of mouse natural killer activity by cholera toxin. Gann..

[188] Anosova N.G., Chabot S., Shreedhar V., Borawski J.A., Dickinson B.L., Neutra M.R. (2008). Cholera toxin, E. coli heat-labile toxin, and non-toxic derivatives induce dendritic cell migration into the follicle-associated epithelium of Peyer's patches. Mucosal Immunol..

[189] Feng B.S., Zheng P.Y., Chen X., Liao X.Q., Yang P.C. (2008). Investigation of the role of cholera toxin in assisting the initiation of the antigen-specific Th2 response. Immunol. Invest..

[190] Krishnamoorthy N., Oriss T.B., Paglia M., Fei M., Yarlagadda M., Vanhaesebroeck B., Ray A., Ray P. (2008). Activation of c-Kit in dendritic cells regulates T helper cell differentiation and allergic asthma. Nat. Med..

[191] Chandy A.G., Nurkkala M., Josefsson A., Eriksson K. (2007). Therapeutic dendritic cell vaccination with Ag coupled to cholera toxin in combination with intratumoural CpG injection leads to complete tumour eradication in mice bearing HPV 16 expressing tumours. Vaccine.

[192] Bimczok D., Rau H., Wundrack N., Naumann M., Rothkötter H.J., McCullough K., Summerfield A. (2007). Cholera toxin promotes the generation of semi-mature porcine monocyte-derived dendritic cells that are unable to stimulate T cells. Vet. Res..

[193] Wendland M., Czeloth N., Mach N., Malissen B., Kremmer E., Pabst O., Förster R. (2007). CCR9 is a homing receptor for plasmacytoid dendritic cells to the small intestine. Proc. Natl. Acad. Sci. USA.

[194] Isomura I., Yasuda Y., Tsujimura K., Takahashi T., Tochikubo K., Morita A. (2005). Recombinant cholera toxin B subunit activates dendritic cells and enhances antitumor immunity. Microbiol. Immunol..

[195] Bagley K.C., Abdelwahab S.F., Tuskan R.G., Lewis G.K. (2004). Calcium signaling through phospholipase C activates dendritic cells to mature and is necessary for the activation and maturation of dendritic cells induced by diverse agonists. Clin. Diagn. Lab. Immunol..

[196] Bagley K.C., Abdelwahab S.F., Tuskan R.G., Fouts T.R., Lewis G.K. (2002). Cholera toxin and heat-labile enterotoxin activate human monocyte-derived dendritic cells and dominantly inhibit cytokine production through a cyclic AMP-dependent pathway. Infect. Immun..

[197] George-Chandy A., Eriksson K., Lebens M., Nordström I., Schön E., Holmgren J. (2001). Cholera toxin B subunit as a carrier molecule promotes antigen presentation and increases CD40 and CD86 expression on antigen-presenting cells. Infect. Immun..

[198] Porgador A., Staats H.F., Itoh Y., Kelsall B.L. (1998). Intranasal immunization with cytotoxic T-lymphocyte epitope peptide and mucosal adjuvant cholera toxin: selective augmentation of peptide-presenting dendritic cells in nasal mucosa-associated lymphoid tissue. Infect. Immun..

[199] Anjuère F., Luci C., Lebens M., Rousseau D., Hervouet C., Milon G., Holmgren J., Ardavin C., Czerkinsky C. (2004). *In vivo* adjuvant-induced mobilization and maturation of gut dendritic cells after oral administration of cholera toxin. J. Immunol..

[200] Gagliardi M.C., De Magistris MT. (2003). Maturation of human dendritic cells induced by the adjuvant cholera toxin: role of cAMP on chemokine receptor expression. Vaccine.

[201] Jank T., Giesemann T., Aktories K. (2007). Rho-glucosylating Clostridium difficile toxins A and B: new insights into structure and function. Glycobiology.

[202] Sandvig K., Spilsberg B., Lauvrak S.U., Torgersen M.L., Iversen T.G., van Deurs B. (2004). Pathways followed by protein toxins into cells. Int. J. Med. Microbiol..

[203] Schirmer J., Aktories K. (2004). Large clostridial cytotoxins: cellular biology of Rho/Ras-glucosylating toxins. Biochim. Biophys. Acta.

[204] Genth H., Dreger S.C., Huelsenbeck J., Just I. (2008). Clostridium difficile toxins: more than mere inhibitors of Rho proteins. Int. J. Biochem. Cell. Biol..

[205] Van Hamme E., Dewerchin H.L., Cornelissen E., Verhasselt B., Nauwynck H.J. (2008). Clathrin- and caveolae-independent entry of feline infectious peritonitis virus in monocytes depends on dynamin. J. Gen.Virol..

[206] Ausiello C.M., Cerquetti M., Fedele G., Spensieri F., Palazzo R., Nasso M., Frezza S., Mastrantonio P. (2006). Surface layer proteins from Clostridium difficile induce inflammatory and regulatory cytokines in human monocytes and dendritic cells. Microbes Infect..

[207] Harokopakis E., Albzreh M.H., Martin M.H., Hajishengallis G. (2006). TLR2 transmodulates monocyte adhesion and transmigration *via* Rac1- and PI3K-mediated inside-out signaling in response to Porphyromonas gingivalis fimbriae. J. Immunol..

[208] Methe H., Kim J.O., Kofler S., Nabauer M., Weis M. (2005). Statins decrease Toll-like receptor 4 expression and downstream signaling in human CD14+ monocytes. Arterioscler. Thromb. Vasc. Biol..

[209] Warny M., Keates A.C., Keates S., Castagliuolo I., Zacks J.K., Aboudola S., Qamar A., Pothoulakis C., LaMont J.T., Kelly C.P. (2000). p38 MAP kinase activation by Clostridium difficile toxin A mediates monocyte necrosis, IL-8 production, and enteritis. J. Clin. Invest..

[210] Jefferson K.K., Smith M.F., Bobak D.A. (1999). Roles of intracellular calcium and NF-kappa B in the Clostridium difficile toxin A-induced up-regulation and secretion of IL-8 from human monocytes. J. Immunol..

[211] Hmama Z., Knutson K.L., Herrera-Velit P., Nandan D., Reiner N.E. (1999). Monocyte adherence induced by lipopolysaccharide involves CD14, LFA-1, and cytohesin-1. Regulation by Rho and phosphatidylinositol 3-kinase. J. Biol. Chem..

[212] Linevsky J.K., Pothoulakis C., Keates S., Warny M., Keates A.C., Lamont J.T., Kelly C.P. (1997). IL-8 release and neutrophil activation by Clostridium difficile toxin-exposed human monocytes. Am. J. Physiol..

[213] Souza M.H., Melo-Filho A.A., Rocha M.F., Lyerly D.M., Cunha F.Q., Lima A.A., Ribeiro R.A. (1997). The involvement of macrophage-derived tumour necrosis factor and lipoxygenase products on the neutrophil recruitment induced by Clostridium difficile toxin B. Immunology.

[214] Siffert J.C., Baldacini O., Kuhry J.G., Wachsmann D., Benabdelmoumene S., Faradji A., Monteil H., Poindron P. (1993). Effects of Clostridium difficile toxin B on human monocytes and macrophages: possible relationship with cytoskeletal rearrangement. Infect. Immun..

[215] Flegel W.A., Müller F., Däubener W., Fischer H.G., Hadding U., Northoff H. (1991). Cytokine response by human monocytes to Clostridium difficile toxin A and toxin B. Infect. Immun..

[216] Kang J.H., Lee G.S., Jeung E.B., Yang M.P. (2009). Trans-10, *cis*-12 conjugated linoleic acid modulates phagocytic responses of canine peripheral blood polymorphonuclear neutrophilic leukocytes exposed to Clostridium difficile toxin B. Vet. Immunol. Immunopathol..

[217] Lee J.Y., Kim H., Cha M.Y., Park H.G., Kim Y.J., Kim I.Y., Kim J.M. (2009). Clostridium difficile toxin A promotes dendritic cell maturation and chemokine CXCL2 expression through p38, IKK, and the NF-kappaB signaling pathway. J. Mol. Med..

[218] El Bekay R., Alba G., Reyes M.E., Chacón P., Vega A., Martín-Nieto J., Jiménez J., Ramos E., Oliván J., Pintado E., Sobrino F. (2007). Rac2 GTPase activation by angiotensin II is modulated by Ca2+/calcineurin and mitogen-activated protein kinases in human neutrophils. J. Mol. Endocrinol..

[219] Brito G.A., Sullivan G.W., Ciesla W.P., Carper H.T., Mandell G.L., Guerrant R.L. (2002). Clostridium difficile toxin A alters *in vitro*-adherent neutrophil morphology and function. J. Infect. Dis..

[220] Servant G., Weiner O.D., Herzmark P., Balla T., Sedat J.W., Bourne H.R. (2000). Polarization of chemoattractant receptor signaling during neutrophil chemotaxis. Science.

[221] Däubener W., Leiser E., von Eichel-Streiber C., Hadding U. (1988). Clostridium difficile toxins A and B inhibit human immune response *in vitro*. Infect. Immun..

[222] Dailey D.C., Kaiser A., Schloemer R.H. (1987). Factors influencing the phagocytosis of Clostridium difficile by human polymorphonuclear leukocy tes. Infect. Immun..

[223] Yanagawa Y., Onoé K. (2002). CCL19 induces rapid dendritic extension of murine dendritic cells. Blood.

[224] Garrett W.S., Chen L.M., Kroschewski R., Ebersold M., Turley S., Trombetta S., Galán J.E., Mellman I. (2000). Developmental control of endocytosis in dendritic cells by Cdc42. Cell.

[225] West M.A., Prescott A.R., Eskelinen E.L., Ridley A.J., Watts C. (2000). Rac is required for constitutive macropinocytosis by dendritic cells but does not control its downregulation. Curr. Biol..

[226] Malorni W., Paradisi S., Dupuis M.L., Fiorentini C., Ramoni C. (1991). Enhancement of cell-mediated cytotoxicity by Clostridium difficile toxin A: an *in vitro* study. Toxicon.

